# Current status of traditional Chinese medicine for the treatment of COVID-19 in China

**DOI:** 10.1186/s13020-021-00461-y

**Published:** 2021-07-27

**Authors:** Liang Chu, Fang Huang, Mengdan Zhang, Biao Huang, Yigang Wang

**Affiliations:** 1 Hepatic Surgery Center, Tongji Hospital, Tongji Medical College, Huazhong University of Science and Technology, No.1095 Jie Fang Avenue, Wuhan, 430030 Hubei China; 2 Department of Pathology, Zhejiang Provincial People’s Hospital, People’s Hospital of Hangzhou Medical College, Hangzhou, 310014 Zhejiang China; 3 College of Life and Medicine Sciences, Zhejiang Sci-Tech University, No. 928, 2nd Street, Xiasha Higher Education Park, Hangzhou, 310018 Zhejiang China

**Keywords:** SARS-CoV-2, COVID-19, Traditional Chinese medicine, Clinical treatment

## Abstract

An ongoing outbreak of severe respiratory illness and pneumonia caused by the severe acute respiratory coronavirus 2 (SARS-CoV-2) commenced in December 2019, and the disease was named as coronavirus disease 2019 (COVID-19). Soon after, scientists identified the characteristics of SARS-CoV-2, including its genome sequence and protein structure. The clinical manifestations of COVID-19 have now been established; and nucleic acid amplification is used for the direct determination of the virus, whereas immunoassays can determine the antibodies against SARS-CoV-2. Clinical trials of several antiviral drugs are ongoing. However, there is still no specific drugs to treat COVID-19. Traditional Chinese medicine (TCM) was used in the treatment of COVID-19 during the early stages of the outbreak in China. Some ancient TCM prescriptions, which were efficacious in the treatment of severe acute respiratory syndrome (SARS) in 2002–03 and the influenza pandemic (H1N1) of 2009, have been improved by experienced TCM practitioners for the treatment of COVID-19 based on their clinical symptoms. These developed new prescriptions include Lianhua Qingwen capsules/granules, Jinhua Qinggan granules and XueBiJing injection, among others. In this review, we have summarized the presenting features of SARS-CoV-2, the clinical characteristics of COVID-19, and the progress in the treatment of COVID-19 using TCMs.

## Introduction

Coronavirus disease 2019 (COVID-19), an acute respiratory infectious disease caused by severe acute respiratory syndrome coronavirus 2 (SARS-CoV-2), was first reported in December 2019 [1]. As of June 16, 2021, over 176,156,662 cases of COVID-19 and more than 3,815,486 deaths have been confirmed worldwide, which includes over 116,665 cases and more than 5306 deaths in China alone [2]. COVID-19 was officially declared a pandemic by the World Health Organization (WHO) on March 11, 2020 [3]. At present, there are no specific drugs to treat COVID-19 [4]. Most treatment schemes proposed by Chinese clinicians stemmed from the extension and improvement of their experience in treating severe acute respiratory syndrome (SARS), middle east respiratory syndrome (MERS) and the influenza pandemic (H1N1) of 2009 earlier this century.

China has a long history of the use of traditional Chinese medicine (TCM) of over 2000 years [5]. This system of treatment has widely spread to Japan, South Korea and other countries [6]. TCMs, including Sang Ju Yin and Yu Ping Feng San among others, have been used in the prevention and treatment of SARS and H1N1 [7]. In a controlled clinical trial [8] performed in 11 hospitals in Hong Kong, China, none of the 1063 health care workers who used the herbal supplement consisting of Sang Ju Yin formula and Yu Ping Feng San formula [9] for two weeks contracted SARS compared to 0.4% of the 36,111 health care workers who did not receive the supplement. During the COVID-19 wave, Chinese clinicians summarized the previous outcomes of the use of TCM in the treatment of viral infections, thoroughly explored several ancient classics formulae, combined their use with clinical practice, and screened six effective prescriptions, namely, Lianhua Qingwen capsules/granules, Jinhua Qinggan granules, XueBiJing injection, Lung Cleansing and detoxifying decoction, Huashibaidu formula and Xuanfeibaidu granules [10]. As of March 23, 74,187 of the confirmed cases of COVID-19 in China, including 61,449 cases in Hubei Province, used TCM [10]. The early clinical effects showed that for mild and moderate patients, it was easy to recover after treatment using TCM; moreover, the progression of the infection from moderate to severe was significantly reduced in patients. In severe and critical patients, TCM therapy could stabilize the blood oxygen saturation, improve dyspnea, and exert a certain auxiliary effect [11]. Whether TCM can be used in the clinical treatment of viral infections depends on two main aspects, namely, the clinical symptoms of patients, and the efficacy of TCM and its previous effects [12]. In this review, we summarize the features of coronaviruses (CoVs), the presenting clinical characteristics of COVID-19, and its treatment using TCMs, which will be helpful to clinicians and medical practitioners.

## Features of CoVs

CoVs are enveloped positive-sense single-stranded RNA viruses [13]. CoVs belong to the Coronavirinae subfamily, Coronaviridae family, and the order Nidovirales. They are divided into four genera, namely, α, β, γ, and δ, among which mainly α and β CoVs infect mammals [14]. CoVs from γ and δ genera mainly infect birds [15]. Prior to 2019, the following six species were known to infect humans: human coronavirus (HCoV)-NL63 and HCoV-229E (belonging to genus α); HCoV-HKU1, HCoV-OC43, SARS-CoV and MERS-CoV (belonging to genus β) [16]. Apart from SARS-CoV and MERS-CoV, the other four HCoVs mainly cause self-limiting diseases [17]. The SARS-CoV and MERS-CoV outbreaks in 2002–03 and 2012, respectively, caused severe acute respiratory illnesses [18]. Currently, SARS-CoV-2 is the seventh HCoV that has been identified, and belongs to the genus β [19].

**Fig. 1:**
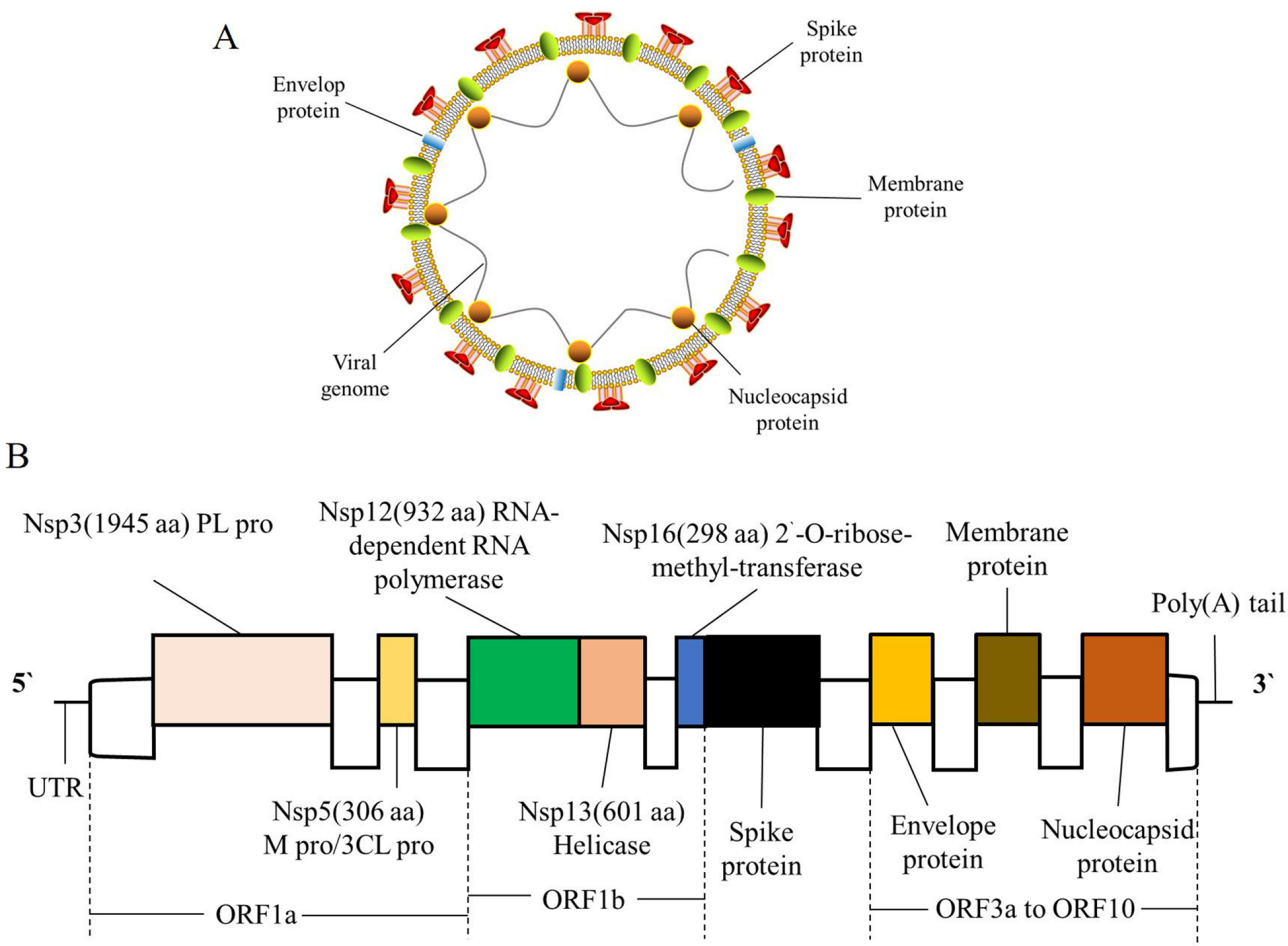
The schematic diagram of severe acute respiratory coronavirus 2 (SARS-CoV-2). **A** The structural proteins of SARS-CoV-2. **B** The map of viral genome of SARS-CoV-2

The size of the CoVs genome ranges from 26–32 Kb, and is currently the largest genome known among RNA viruses [20]. It contains 7–10 different open reading frames (ORFs) with methylation at the 5ʹ cap structure and 3ʹ polyadenylation [20]. CoVs contain four major structural proteins, namely, the spike (S), membrane (M), envelope (E), and nucleocapsid (N) protein, as well as a number of accessory ORF proteins [21]. The S protein is a large oligomeric transmembrane protein responsible for receptor binding and cell fusion [21]. The M protein participates in budding and envelope formation, and plays a key role in virus assembly [22]. SARS-CoV-2 shares 76.7%, 33.8% and 96.2% overall genome sequence identity with SARS-CoV, MERS-CoV and bat CoV RaTG13, respectively [18]. A structural study of the S protein suggests that the SARS-CoV-2 S protein retains sufficient affinity to the human angiotensin-converting enzyme 2 (ACE2) protein [23], and uses ACE2 protein as the primary receptor for cellular entry (Fig. 1), as that for SARS-CoV S protein [24].

## Clinical characteristics of COVID-19

### COVID-19 symptoms

Huang et al. [25] reported that the common symptoms were fever (98%), cough (76%), and myalgia or fatigue (44%) at the onset of the infection, based on the presentation of 41 COVID-19 patients in Wuhan by Jan 2, 2020. Sputum production (28%), headache (8%), hemoptysis (5%), and diarrhea (3%) were rare. All 41 patients had pneumonia and abnormal imaging patterns, which were observed in chest computerized tomography (CT) scans. The plasma concentrations of interleukin (IL) 2, IL7, IL10, granulocyte-colony stimulating factor (G-CSF), IFN-γ-inducible protein 10 (IP-10/CXCL10), monocyte chemoattractant protein-1 (MCP-1/CCL2), macrophage inflammatory protein-1α (MIP-1α/CCL3), and tumor necrosis factor-α (TNF-α) were higher in patients in the intensive care unit (ICU) than that of non-ICU patients. The report published by Guan et al. [26] was a retrospective study comprising 1099 patients with laboratory-confirmed COVID-19, which found that the most common symptoms were fever (43.8% on admission and 88.7% during hospitalization) and cough (67.8%); while vomiting (5.0%) and diarrhea (3.8%) were rare. About 23.7% of patients had at least one coexisting illness (e.g., hypertension or chronic obstructive pulmonary disease) and 86.2% (840/975) patients on admission were found to have abnormalities based on the results of their CT scan. Among them, 56.4% of chest CT scans reveals a ground-glass opacity pattern, while 51.8% had bilateral patchy shadowing. In addition, there were many asymptomatic patients who did not exhibit fever, fatigue or other clinical manifestations, other than positive nucleic acid or antibody tests. Such asymptomatic patients can also spread infections. Since their condition is stable and the viral load is relatively low, the transmission ability is relatively weak [27].

### Diagnosis of COVID-19

As the symptoms of COVID-19 and other viral pneumonia may overlap, nucleic acid detection using real-time polymerase chain reaction (RT-PCR), is considered essential in the diagnosis [28]. However, false-negative RT-PCR test results [29] lead to inconsistencies between clinical symptoms and findings of lung imaging. Additionally, the process of RT-PCR is time consuming (usual 2–3 h). Compared to RT-PCR, CT imaging for chest is more popular and accurate, and has the added advantage of the ease of operation [30]. Moreover, it can be used to obtain near real-time images, helping medical practitioners promptly assess the lung condition of patients. However, CT imaging may not be a useful tool to differentiate between COVID-19 and other viral pneumonia; thus it cannot completely replace nucleic acid detection [27, 31]. It is well known that immunoglobulin (Ig) M is produced in the early stages of viral infections, which can indicate current or the most recent infection [32]. IgG is the main antibody response for long-term immunity and immunological memory, and indicates that either the disease is in its recovery period or there is a prior infection [32]. Therefore, the presence of the IgM and IgG antibodies could likely indicate a SARS-CoV-2 infection [33]. Several clinical test statistics show that the combined IgM-IgG antibody detection can effectively reduce false-negative results during nucleic acid detection [34]. However, there are some false-positive results while performing an IgM-IgG immunoassay because (1) some weak positive results near the Cut-off value are likely to be misinterpreted as false positive; (2) the presence of endogenous or exogenous interfering substances in patient samples could lead to false-positive results [35].

## Treatment of COVID-19 with TCM

So far, no therapeutic agents have been proven to be effective for SARS-CoV-2-caused diseases [36]. TCM is based on the central premise that when pathogens invade the body to cause diseases, they reduce the immune function of the body [37]. In general, the reduced immune function is further weakened if only simple anti-pathogen treatment is used. TCM improves immune function by improving the body’s resistance to disease, physical conditions and by reducing the side effects of Western medicine [38]. Based on China’s experience in using TCM for more than 2000 years and its success in the treatment of patients during the 2002–03 SARS and 2009 H1N1 epidemics [39–41], some TCM prescriptions have been rapidly developed and used for the management of COVID-19 [42–46]. Moreover, some bioactive components, such as glycyrrhizic acid, a component of licorice [47], baicalin, a flavonoid [48], and ginsenosides [49], have been reported to inhibit the replication of SARS-CoV, indicating their potential in the treatment of COVID-19.

### Lianhua Qingwen (LH) capsules/granules

Lianhua Qingwen (LH) is a Chinese patent medicine produced by the reduction of a combination of Yinqiao San and Maxing Shigan decoctions [50]. Maxing Shigan decoction was originally described in a classic Chinese medicine book, Shanghan Lun written by Zhang Zhongjing of the Eastern Han Dynasty about 1800 years ago [51]. Its ingredients include Ephedra sinica Stapf (6 g), Prunus amygdalus Batsch (9 g), Gypsum (24 g) and Glycyrrhiza glabra L. (6 g). Yinqiao San is a prescription from the TCM monograph,Wen bing Tiao bian, of the Qing Dynasty about 300 years ago [52]. Its main ingredients include *Forsythia suspensa* (Thunb.) Vahl (30 g), *Lonicera japonica* Thunb. (30 g), *Platycodon grandiflorus* (Jacq.) A.DC. (18 g), *Mentha canadensis* L. (18 g), Bamboo leaf (12 g), *Glycyrrhiza glabra* L. (15 g), *Brassica juncea* (L.) Czern. (12 g), Tempeh (15 g) and *Arctium lappa* L. (18 g). LH is a prescription formulated under the TCM guidance of collateral disease theory and the treatment method of “clearing away plague and detoxification”, and composed of 13 herbs [53], including *Isatis tinctoria* L. (Banlangen), *Forsythiae fructus* (Lianqiao), *Lonicera japonica* Thunb. (Jinyinhua), *Dryopteris crassirhizoma* Nakai (Mianmaguanzhong), *Ephedra sinica* Stapf (Mahuang), *Prunus armeniaca* L. (Kuxingren), *Houttuynia cordata* Thunb. (Yuxingcao), *Pogostemon cablin* (Blanco) Benth. (Guanghuoxiang), *Rhodiola crenulata* (Hook.f. & Thomson) *H. Ohba* (Hongjingtian), *Rheum officinale* Baill. (Dahuang), *Glycyrrhiza inflata* Batalin (Gancao), Gypsum Fibrosum (Shigao), and l-Menthol (Bohenao) (Table 1).

**Table 1:** The composition, indications and side effects of 6 prescriptions in COVID-19 treatment

Name	Herbal composition	Indications	Side effects
Lianhua Qingwen capsule/granule	*Isatis tinctoria* L. (Banlangen), *Forsythiae Fructus* (Lianqiao), *Lonicera japonica* Thunb. (Jinyinhua), *Dryopteris crassirhizoma* Nakai (Mianmaguanzhong), *Ephedra sinica* Stapf (Mahuang), *Prunus armeniaca* L. (Kuxingren), *Houttuynia cordata* Thunb. (Yuxingcao), *Pogostemon cablin* (Blanco) Benth. (Guanghuoxiang), *Rhodiola crenulata* (Hook.f. & Thomson) *H. Ohba* (Hongjingtian), *Rheum officinale* Baill. (Dahuang), *Glycyrrhiza inflata* Batalin (Gancao), *Gypsum Fibrosum* (Shigao), l-menthol (Bohenao)	Fever or high fever, chills, muscle soreness, stuffy nose, runny nose, cough, headache, dry throat, sore throat, red tongue, yellow tongue coating	Nausea, poor appetite, abdominal distention, itchy skin
Jinhua Qinggan granule	*Lonicera japonica* Thunb. (Jinyinhua), *Gypsum Fibrosum* (Shigao), *Ephedra sinica* Stapf (Mahuang), *Prunus armeniaca* L. (Kuxingren), *Scutellaria baicalensis* Georgi (Huangqin), *Forsythia suspensa* (Thunb.) Vahl (Lianqiao), *Fritillaria thunbergii* Miq. (Zhebeimu), *Anemarrhena asphodeloides* Bunge (Zhimu), *Arctium lappa* L. (Niubangzi), *Artemisia annua* L. (Qinghao), *Mentha canadensis* L. (Bohe), *Glycyrrhiza inflata* Batalin (Gancao)	Mild influenza symptoms with fever, headache, muscle pain, sore throat, cough, stuffy nose and runny nose	Nausea, vomit, diarrhea, stomach discomfort. Seldom: abnormal liver function, heart palpitation and rash
XueBiJing injection	*Paeonia lactiflora* Pall. (Chishao), Conioselinum anthriscoides ‘Chuanxiong’ (Chuanxiong), *Salvia miltiorrhiza* Bunge (Danshen), *Carthamus tinctorius* L. (Honghua), *Angelica sinensis* (Oliv.) Diels (Danggui)	Fever, shortness of breath, heart palpitation, restlessness	Itchy skin
LungCleansing and detoxifying decoction	*Ephedra sinica* Stapf (Mahuang) 9 g, Roasted *Glycyrrhiza glabra* L. 6 g, *Prunus amygdalus* Batsch 9 g, Raw Gypsum (decocting first) 15–30 g, Guizhi 9 g, *Alisma plantago-aquatica* L. 9 g, Polyporus 9 g, *Atractylodes macrocephala* Koidz. (Baizhu) 9 g, Poria Cocos 15 g, *Bupleurum chinense* DC. (Chaihu) 16 g, *Scutellaria baicalensis* Georgi 6 g, *Pinellia ternata* 9 g, *Zingiber officinale* Roscoe 9 g, *Aster tataricus* L.f. 9 g, *Tussilago farfara* L. (Donghua) 9 g, *Iris domestica* (L.) Goldblatt & Mabb. (Shegan) 9 g, *Asarum sieboldii* Miq. 6 g, *Dioscorea alata* L. 12 g, Zhishi 6 g, Chenpi 6 g, *Pogostemon cablin* (Blanco) Benth. (Huoxiang) 9 g	Mild, moderate, severe and critical patients. Prescription for disease treatment, not recommended for prevention	N/A
Huashibaidu formula	*Ephedra sinica* Stapf 6 g, *Prunus amygdalus* Batsch 9 g, Gypsum 15 g, *Paeonia lactiflora* Pall. 10 g, *Ageratum conyzoides* L. 10 g, Lepidium Seed 10 g, *Glycyrrhiza glabra* L. 3 g, *Pinellia ternata* 9 g, Poria Cocos 15 g, Fructus Tsaoko 10 g, Cablin Atractylodes 15 g, *Astragalus mongholicus* Bunge 10 g, *Magnolia Officinalis* 10 g, *Rheum officinale* Baill. 5 g	Light, moderate and severe patients	N/A
Xuanfeibaidu granule	*Ephedra sinica* Stapf 6 g, Bitter Apricot Kernel 15 g, Gypsum 30 g, Raw Coix Seed 30 g, *Atractylodes lancea* 10 g, *Pogostemon cablin* (Blanco) 15 g, *Artemisia abrotanum* L. 12 g, *Reynoutria japonica* Houtt. 20 g, *Verbena officinalis* L. 30 g, Dried *Phragmites australis* 30 g, *Lepidium didymum* L. Seed 15 g, Citrus maxima 15 g, Raw *Glycyrrhiza glabra* L. 10 g	Light and moderate patients	N/A

**Fig. 2:**
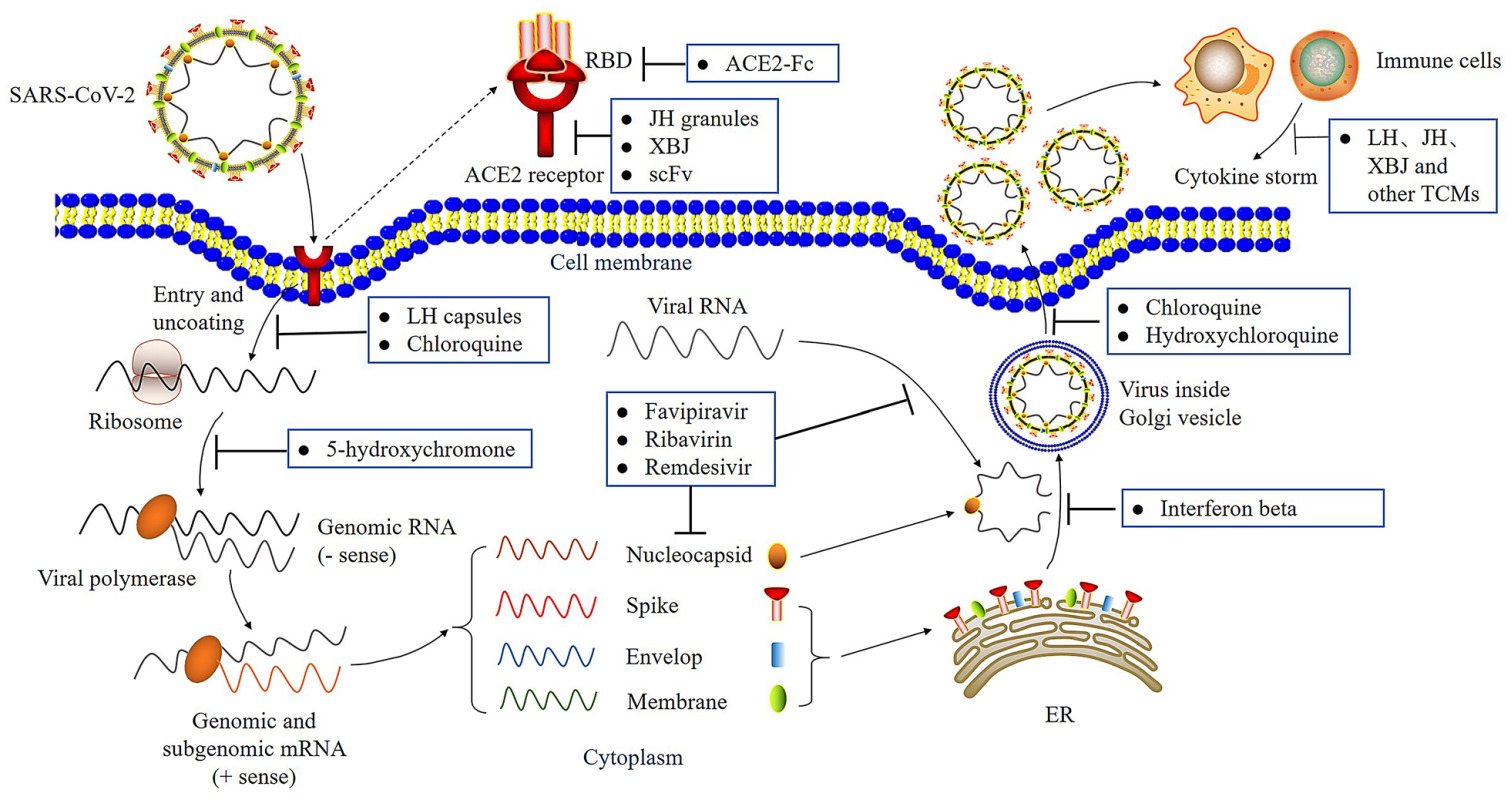
The replication cycle of SARS-CoV-2 and the targets of various therapeutic drugs. SARS-CoV-2 recognizes the ACE2 receptor on the host cell membrane via the RBD of spike protein, and enters host cell. Once uncoating, the viral genomic RNA will be used to synthesize the genomic RNA (- sense). Next, the genomic RNA is used as a template to form genomic and subgenomic mRNA (+ sense). The viral RNA and nucleocapsid protein are synthesized in the cytoplasm, while the viral spike, envelop, membrane proteins are transcribed and translated in the ER. After that, the SARS-CoV-2 are assembled to form mature virion and encapsulated in the Golgi vesicle. Viruses are released outside the host cell membrane via vesicle-mediated exocytosis, and further activate host immune system. *RBD* receptor-binding domain, *ACE2* angiotensin-converting enzyme 2, *Fc* immunoglobulin fragment, *scFv* single-chain variable region fragments against spike protein of SARS-CoV2, *ER* endoplasmic reticulum

LH has been recommended for the treatment of influenza A viral infection [54] and influenza complicated with bronchial pneumonia in humans [55]. LH exerts a broad-spectrum antiproliferative effect against influenza viruses, including those that caused the H1N1 and H7N9 pandemics. LH exerts its effects by blocking the early cell entry of the virus in the lung, and suppresses the virus-induced NF-kB activation and release of the inflammatory cytokines, including IL-6, TNF-α, and MCP-1, thereby particularly improving the immune response to prevent viral infection [56] (Fig. 2). Besides, the role of LH in the treatment of 2002–03 SARS in China [57] preliminarily affirmed its efficacy in the management of coronavirus infection. In addition, LH is efficacious in the animal models of MERS.

Dong et al. [58] investigated the effects of LH on airway inflammation in 100 patients with acute exacerbation of chronic obstructive pulmonary disease. They reported that LH treatment decreased the expression of IL-8, TNF-α, IL-17, and IL-23 in the sputum, and of IL-8 and IL-17 in the blood, suggesting that the mechanism of action of LH was related to the decreased release of the inflammatory mediators. A retrospective, not double blind study comprising 42 patients with COVID-19 in three hospitals in Wuhan was performed to compare the effect of the combination of LH plus standard care (21 patients) and standard care alone (21 patients). The result showed that the disappearance rate of fever (85.7% vs 57.1%), cough (46.7% vs 5.6%), expectoration (64.3% vs 9.1%), and shortness of breath (77.8% vs 0) in the combined treatment group was better (P < 0.05) than that in the control group. The duration of fever in the combined treatment group was 1.5 days shorter than that in the control group (4.6 ± 3.2 vs 6.1 ± 3.1) [59]. Another multicentre retrospective, not double blind study compared the clinical data of 51 patients receiving LH combined with standard care and 51 receiving standard care alone in three hospitals in Wuhan during January 2020. The combined treatment showed a better (P < 0.05) therapeutic effect than the control in the disappearance rate of fever (83.7% vs 61.0%), fatigue (61.3% vs 34.3%), cough (62.2% vs 35.9%), expectoration (55.0% vs 15.8%), shortness of breath (61.5% vs 14.3%), chest distress (54.6% vs 15.8%), anorexia (34.8% vs 7.7%), and the rate of progressing to severe symptoms (7.8% vs 21.6%). There was no difference in lung improvement rate (54.9% vs 45.1%, P > 0.05) [60]. A prospective, multicentre, open-label randomized controlled trial evaluating LH capsules in 284 confirmed cases of COVID-19 was performed [61]. Patients were randomized to receive standard treatment alone (142 patients) or a combination of standard treatment and LH capsules (4 capsules, thrice daily, 142 patients) for 14 days. The combined treatment group exhibited a higher recovery rate than the control group (91.5% vs 82.4%, P = 0.022). The median time to symptom recovery, recovery from fever, fatigue, and cough was significantly shorter in the combined treatment group versus the control group (median: 7 vs 10 days, 2 vs 3 days, 3 vs 6 days, 7 vs 10 days, respectively, all P ≤ 0.001). The recovery rate of abnormal manifestations within the chest evaluated by chest CT (83.8% vs 64.1%, P ≤ 0.001) and clinical cure (78.9% vs 66.2%, P = 0.017) was markedly higher in the treatment group compared to the control group, respectively. There was no difference in the rate of conversion to severe cases and in the median viral assay conversion time between the two groups. These results indicated that LH was effective in improving fever, cough, and other symptoms in patients with a mild infection of COVID-19. A meta-analysis of 154 COVID-19 patients showed that the disappearance rate of the main clinical symptoms (fever, cough and fatigue) was higher in LH treated group, and the disappearance rate of other clinical secondary symptoms (runny nose, sputum, nasal congestion, muscle pain, difficulty breathing, chest tightness, nausea and vomiting, and loss of appetite) was also higher, while the duration of fever was significantly reduced by LH treatment [62]. In addition, results from a recent meta-analysis of 8 clinical trials with 924 COVID-19 patients indicated that patients treated by LH combined with conventional treatment (e.g. oxygen therapy, antiviral, antimicrobial) had a higher overall effective rate and CT recovery rate than those with conventional treatment [63].

The antiviral activity of LH in SARS-CoV-2 was studied using Vero E6 cells. LH significantly inhibited the replication of SARS-CoV-2 obtained from a clinical isolated and reduced mRNA levels of the pro-inflammatory cytokines, including TNF-α, IL-6, MCP-1/CCL2, and IP-10/CXCL10, suggesting that LH might inhibit the cytokine storm induced by SARS-CoV-2 [43]. According to a recent study published on ChemRxiv [64], 20 components in LH were identified using ultra-performance liquid chromatography coupled to quadrupole time of flight mass spectrometry. The active components of LH include rutin, forsythoside E, hyperoside, liquiritin apioside, emodin, chlorogenic acid, amygdalin, cryptochlorogenic acid, isoliquiritin apioside, neochlorogenic acid, chrysophanol-8-*O*-glucoside, rhein, isoliquiritin, emodin 8-*O*-β-d-glucoside, sweroside, formononetin, salidroside, liquiritigenin, loqanic acid, and secolologanin, among others. Among them, three compounds, namely hyperoside, rutin, and forsythoside, were found to be bound with the main protease of SARS-CoV-2 and had docking scores of at − 9.1, − 9.0 and − 8.7 kcal/mol, respectively, which were better than that of lopinavir (− 7.3 kcal/mol). Using the component-target-pathway network analysis, the authors indicated that LH alleviates the symptoms of COVID-19 by activating the antiviral and anti-inflammatory responses of cells. LH has been recommended by the Evidence-Based Medicine Chapter of China International Exchange and Promotive Association for Medical and Health Care (CPAM) to treat patients with mild or moderate COVID-19 in combination with conventional therapy [65], and some clinical evidences showed the conjunction therapy could improve COVID-19 patients, suggesting LH would be beneficial as a supplymentary strategy for treating COVID-19.

### Jinhua Qinggan (JH) granules

JH was developed during the H1N1 epidemic in 2009 [66]. This prescription is composed of two prescriptions, namely Maxing Shigan decoction and Yinqiao San, and is similar to LH [67]. JH comprises 12 herbs, including *Lonicera japonica* Thunb. (Jinyinhua), *Gypsum Fibrosum* (Shigao), *Ephedra sinica* Stapf (Mahuang), *Prunus armeniaca* L. (Kuxingren), *Scutellaria baicalensis* Georgi (Huangqin), *Forsythia suspensa* (Thunb.) *Vahl* (Lianqiao), *Fritillaria thunbergii* Miq. (Zhebeimu), *Anemarrhena asphodeloides* Bunge (Zhimu), *Arctium lappa* L. (Niubangzi), *Artemisia annua* L. (Qinghao), *Mentha canadensis* L. (Bohe), and *Glycyrrhiza inflata* Batalin (Gancao) [68]. Molecular docking analysis showed that the key compounds of JH, namely formononetin, stigmasterol, β-sitosterol, and anhydroicaritin, had a certain degree of affinity to SARS-CoV-2 3CL hydrolase (Mpro) and ACE2 [69, 70]. Another study by Simayi et al. [71]. indicated that the active components, kaempferol, baicalein, and oroxylin A of JH granules had a high affinity to the M protein of SARS-CoV-2, and were related to the anti-inflammatory response and apoptotic signaling pathways by binding to ACE2 to exert a therapeutic effect in COVID-19.

JH is efficacious in treating mild and moderate COVID-19 patients, helps restore the lymphocyte and white blood cell count, and reduces the rate of mild patients worsening to the severe form of the infection [72]. A recently concluded clinical study performed in Wuhan showed that the disappearance rate of the symptoms of fever (80.3% vs 53.1%), cough (66.1% vs 42.9%), fatigue (77.6% vs 53.8%), and expectoration (85.3% vs 46.2%) in the treatment group was significantly higher than that in the control group. The study compared 82 patients with mild COVID-19 treated with JH combined with standard care to 41 mild COVID-19 patients treated with standard care alone [73]. Another retrospectively, not double blind clinical trial has also been performed to evaluate the efficacy and safety of JH in 80 patients with COVID-19 in Beijing. The results indicated that the average duration of viral nucleic acid detection (test negative) and the pneumonia recovery time were shorter in JH treatment group (7 ± 4) d and (8 ± 4) d than that control group (10 ± 4) d and (10 ± 5) d, P = 0.010 and P = 0.021, respectively. Further, the 7-day viral clearance rate was significantly higher in the JH group (56.82%) compared with the control group (27.78%, P = 0.009), and no adverse effects existed in the JH treatment [74]. Based on the experience of clinicians, patients with mild fever and severe headache should be treated with JH, while those with high fever and dry stools should be treated with LH [72]. Shi et al. [75] found that both LH and JH had multiple antiviral activities by targeting viral life cycle and regulating host immune responses and inflammation through literature mining. Moreover, JH is more potent in modulating viral life cycle, whereas LH exhibits better efficacy in regulating host anti-viral responses, suggesting LH and JH could be potentially an alternative therapy for emerging viral diseases. They also found that both LH and JH are potentially being able to prevent the progress of COVID-19 into severe or critical conditions. Oral administration of LH could be more beneficial for patients with insufficient immune functions or for patients with alleviated symptoms after treatment with JH.

### Xuebijing (XBJ) injection

XBJ was developed during the SARS epidemic in 2002–03 [76]. It is a Chinese herbal-based therapeutic injection consisting of *Paeonia lactiflora* Pall. (Chishao), *Conioselinum anthriscoides* “Chuanxiong” (Chuanxiong), *Salvia miltiorrhiza* Bunge (Danshen), *Carthamus tinctorius* L. (Honghua), and *Angelica sinensis* (Oliv.) Diels (Danggui). Ultra-high-performance liquid chromatography coupled with quadrupole Orbitrap high-resolution mass spectrometry (UHPLC-Q-Orbitrap HRMS) indicates that the primary components of XBJ are hydroxysafflor yellow A, oxypaeoniforin, senkyunolide I, and benzoylpaeoniflorin [77]. XBJ has been approved for use in the treatment of severe infections (sepsis) in critically ill patients (China Food and Drug Administration; Beijing, China, Number Z20040033). A prospective, randomized, controlled study on XBJ injection for critically ill patients with severe community-acquired pneumonia was performed in 33 hospitals in China [78]. Results from the study indicate that XBJ injection significantly improves the primary endpoint of the pneumonia severity index as well as brings about a significant improvement in the secondary clinical outcomes of mortality, reduces the duration of mechanical ventilation, and shortens the duration of ICU stay [78]. Chen et al. [79] showed that XBJ facilitates the expansion of IL-10^+^ Tregs and normalizes the pro-inflammatory Th17 population in mice with sepsis. XBJ also reduces the levels of the cytokines, TNF-α and IL-6, which are known to participate in the inflammatory response. Liu et al. [80] report that XBJ significantly improves the survival rate of mice with sepsis by promoting M2 polarization of the macrophages, thereby contributing to a therapeutic effect in sepsis and providing the mechanism for further treatment of COVID-19.

The observation in 11 severe or critical COVID-19 patients received XBJ treatment showed that the expression of TNF-α, IP-10, macrophage inflammatory protein-1β (MIP-1β) and RANTES protein was inhibited during day 7–8 of treatment. In addition, XBJ has been shown to protect Vero E6 cells from SARS-CoV-2-induced cell death[81]. A randomized, double-blinded trial with 60 COVID-19 patients (3 dropped out) indicated that the secretion of IL-6, IL-8 and TNF-α was significantly suppressed in the routine medication plus XBJ therapy group [82]. After 14 days of treatment, the lymphocyte level was higher, and the C-reactive protein level was lower in the combination therapy group than the routine medication plus saline group [82]. However, there was no difference between the 28-day mortality of the two groups. XBJ combined with routine treatment significantly reduced IL-6 levels and body temperature in a retrospective case–control study with 42 COVID-19 patients [83]. XBJ was recommended by China's National Health Commission to treat severe and critical cases of COVID-19, especially during systematic inflammatory response syndrome (SIRS) and/or multi-organ failure [84, 85].

### Other TCMs in the treatment of COVID-19

Lung Cleansing and detoxifying decoction is composed of four compound prescriptions, i.e. Maxing shigan decoction, Shegan Mahuang decoction, Xiaochaihu decoction, and Wuling San, comprising a total of 21 herbal components [86]. It is mainly effective in reducing the symptoms of fever, cough, and fatigue, as well as in improving the lung condition in severe patients. It is a prescription for disease treatment and is not recommended to be used as a prophylactic [87]. Xuanfeibaidu granules (XFBD) are composed of four prescriptions, namely, Maxing shigan decoction, Maxingyigan decoction, Qianjinweigan decoction, and Tingledazaoxiefeng decoction [88], and is currently in the process to obtain permission for new drug research. It is effective in improving symptoms of COVID-19, including fever, cough, suffocation, and fatigue, especially when combined another TCM, Reduning injection [89]. In addition, mechanically XFBD has an obvious effect in reducing the levels of C-reactive protein, increasing the lymphocyte count, balancing immunity, eliminating inflammation, regulating and recovering metabolism [90]. Huashibaidu formula is composed of 14 herbal components and is approved for clinical trials as a new drug [91]. Moreover, according to the 7th trial version of “Diagnosis and Treatment Protocol for Novel Coronavirus Pneumonia”, Huoxiang Zhengqi capsules/oral solution and Shufeng Jiedu capsules/granules were suggested to use in medical observation period of COVID-19; Xiyanping injection, Reduning injection, Tanreqing injection, and Xingnaojing injection were also suggested to use in severe cases; and Reduning injection, Tanreqing injection, Xingnaojing injection, Shenfu injection, and Shengmai injection was used in critical cases [11, 85]. In addition, there are nine TCMs that have been approved for national clinical trials (Table 2).

**Table 2:** Clinical trials of TCMs for prevention and treatment of COVID-19

No	Study Title	TCM Drug	Status	Phase	NCT_ID/registration No	Study type	Applicant’s institution
1	Yinhu Qingwen decoction for the treatment of mild/common COVID-19	YinHu QingWen Decoction	Not yet recruiting	Phase 2 Phase 3	NCT04278963	Interventional study	Jingzhou Hospital of Traditional Chinese Medicine
2	Yinhu Qingwen Granula for the treatment of severe COVID-19	Yinhu Qingwen Granula	Not yet recruiting	Phase 2 Phase 3	NCT04310865	Interventional study	Wuhan No.7 Hospital/Jizhong Energy Fengfeng Group Hospital
3	Clinical trial on regularity of TCM syndrome and differentiation treatment of COVID-19	TCM prescriptions: Huoxiang, Suye, Cangzhu, Houpo, Qianhu, Chaihu, Huangqin, Qinghao, Xingren, JInyinhua, Lianqiao	Not yet recruiting	N/A	NCT04306497	Interventional study	Huai'an fourth people's Hospital
4	Treatment of pulmonary fibrosis due to 2019-nCoV pneumonia with Fuzheng Huayu	Fuzheng Huayu Tablet + *N*-acetylcysteine	Not yet recruiting	Phase 2	NCT04279197	Interventional study	Shuguang Hospital, Shanghai, China
5	A randomized, open-label, blank-controlled trial for Lian-Hua Qing-Wen Capsule/Granule in the treatment of novel coronavirus pneumonia (COVID-19)	Lianhua Qingwen Capsule/Granule	Recruiting	Phase 4	ChiCTR2000029434	Interventional study	Hebei Yiling Hospital, Renmin Hospital of Wuhan University
6	Efficacy and safety of Xue-Bi-Jing injection in the treatment of severe cases of novel coronavirus pneumonia (COVID-19)	Xuebijing injection	Recruiting	Phase 0	ChiCTR2000030388	Interventional study	Jingzhou First People's Hospital
7	Retrospective study for the efficacy of ulinastatin combined with “clear lung detoxification soup” in the treatment of novel coronavirus pneumonia (COVID-19)	LungCleansing and detoxifying decoction	Recruiting	Phase 1	ChiCTR2000030806	Interventional study	Wuhan 3rd Hospital
8	Clinical research and preparation development of qingfei detoxification decoction (mixture) for prevention and treatment of novel coronavirus pneumonia (COVID-19)	LungCleansing and detoxifying decoction	Recruiting	N/A	ChiCTR2000030883	Interventional study	Affiliated Hospital of Traditional Chinese Medicine, Southwest Medical University
9	A randomized controlled trial for Hua-Shi Bai-Du granules in patients with novel coronavirus pneumonia (COVID-19)	Huashibaidu Formula	Recruiting	N/A	ChiCTR2000030988	Interventional study	Guangdong Provincial Hospital of Chinese Medicine

## Therapy of COVID-19 with modern medicine and vaccine

The present clinical methods for COVID-19 treatment mainly include antiviral and antibacterial drugs, anti-inflammatory drugs, anti-SARS-CoV-2 antibody, oxygen therapy, and intestinal microecological agents and plasma from patients in rehabilitation [92, 93]. It is widely understood that patients in the early stages of COVID-19 may benefit from antiviral drugs to reduce viral replication, while patients in severe or advanced stages may benefit from anti-inflammatory treatment [94].

The guidelines for the diagnosis and treatment of novel coronavirus pneumonia (the seventh edition) issued by the National Health Commission and the State Administration of TCM of the People’s Republic of China recommends the use of certain antiviral drugs, but not the anti-COVID-19 indications, such as the HIV-protease inhibitors lopinavir and ritonavir, and the broad-spectrum antiviral drug, ribavirin [36, 95, 96]. However, a randomized, controlled, open-label trial on lopinavir and ritonavir showed that the combination was not beneficial in 99 COVID-19 patients who were in a critical condition compared to the 100 serious patients who were receiving standard care to accelerate clinical improvement, reduce mortality, and reduce the viral RNA detectability from the throat. The efficacy of the combination of lopinavir and ritonavir and other antiviral drugs in COVID-19 remains to be determined [97]. Remdesivir was originally tested in Ebola-infected patients [98]. It previously received emergency use authorization (EUA) from the Food and Drug Administration (FDA) in May 2020, for use in patients with severe COVID-19. In August, the FDA relaxed its guidelines to extend the use of the drug in less serious cases. In October, the FDA gave full approval to remdesivir for the treatment of COVID-19 [99]. However, preliminary results from the Solidarity Trial by the WHO showed that remdesivir had no effect on mortality and had negligible outcomes on the length of hospital stay [100]. Moreover, umifenovir and favipiravir, both for influenza prophylaxis, have also been used in COVID-19 treatment, and the latter exerted the better clinical effect for moderate COVID-19 patients in a prospective randomized study [101]. It is worth mentioning that chloroquine/hydroxychloroquine, used for malaria and rheumatoid arthritis, have also been applied to treat COVID-19 in clinic, although their efficacy in inhibiting pneumonia progression and replication of SARS-CoV-2 needs to be further considered [97, 102]. On September 2, 2020, the WHO issued an interim guideline on the use of dexamethasone and other corticosteroids for the treatment of COVID-19. Those drugs are used in the management of several conditions, owing to their anti-inflammatory and immunosuppressant effects. The WHO strongly recommends the oral or intravenous administrations of corticosteroids in patients with severe and critical COVID-19 and advises against its use in those with non-severe COVID-19, unless the patient condition[103] (Fig. 2). In addition, many novel therapeutics, including convalescent plasma treatment, mono-antibody bamlanivimab derived from plasma, antibody against inflammatory (Tocilizumab), interferon-α/-β and stem cell therapy, etc. are also used or ongoing in the clinical treatment of COVID-19 [104]. More importantly, many teams worldwide have employed multiple strategies to develop vaccines against COVID-19, which need to undergo stringent clinical trials, as in the case of drugs [105]. These vaccines include nucleic acid vaccine (DNA or RNA), adenovirus vaccine, inactivated virus vaccine, attenuated live virus vaccine and recombinant protein vaccine. Presently, more than 287 vaccines are developed around the world at different stages according to WHO reports, and 102 vaccines are already in clinical use, which will build an effective barrier against COVID-19 (Table 3).

**Table 3:** Candidate vaccines for COVID-19 (as of June, 2021)

Vaccine platform description	Candidate vaccines in clinical phase (No.)	Candidate vaccines in pre-clinical phase (No.)
Protein subunit	32	72
Viral vector (non-replicating)	16	21
Inactivated virus	16	9
RNA based vaccine	16	24
DNA based vaccine	10	16
Virus like particle	5	18
Viral vector (replicating)	2	19
Viral vector (replicating) + antigen presenting cell	2	0
Live attenuated virus	2	2
Live attenuated bacterial vector	0	2
Viral vector (non-replicating) + antigen presenting cell	1	0
Bacterial vector (Replicating)	0	1
Cellular based vaccine	0	1
Total	102	185

Source: WHO

## Conclusion

Currently, COVID-19 pandemic is still rapidly spreading around the worldwide. The genome sequence, protein structure and the features of SARS-CoV-2 were identified since the finding of SARS-CoV-2. The diagnoses of COVID-19 have made a great progress through nucleic acid amplification for the virus genome and immunoassay for the antibodies against SARS-CoV-2. Although COVID-19 has been propagated around the world for more than a year, there are still few effective drugs to prevent it. Fortunately, the worldwide vaccination of SARS-CoV-2 has brought hope for the prevention and elimination of COVID-19.

TCM is a whole set of treatment methods and prescriptions based on individual syndrome differentiation. It pursues to achieve the balance of Yin and Yang in the human body. It resists external infections by adjusting the overall immunity. Compared to Western medicine, which mostly uses chemical drugs with single and specific targets, TCM primarily uses compound plant formulations and mixtures with numerous and miscellaneous targets [106]. Due to the successful experience in treating SARS of 2002–03 and the H1N1 influenza pandemic of 2009 using ancient TCM prescriptions, TCM was rapidly applied to treat COVID-19 and attained effective results since the early outbreak of COVID-19 in China. Moreover, new TCM prescriptions have been quickly developed according to the clinical symptoms of COVID-19 patients. Among them, TCM “three medicines three parties” play a crucial role in against COVID-19 in China, which including “Three medicines”-Lianhua Qingwen capsules/granules, Jinhua Qinggan granules and XueBiJing injection, and “Three parties”-LungCleansing and detoxifying decoction, Huashibaidu formula, Xuanfeibaidu granule. Since then, the molecular mechanism of TCM against COVID-19 has been reported constantly.

## Limitations of TCM and prospects for future research

It exists on the limitations for TCM treatment of COVID-19. For example, it is very difficult to maintain the stability of the various formulations of TCM. The time and method of decocting can alter the composition of the final product. At present, only limited data on COVID-19 cases are available. Therefore, additional prospective cohort studies and randomized controlled trials are needed to evaluate the efficacy of TCM in preventing and treating COVID-19, as well as to explore and identify suitable methods to combine TCM and Western medicine [107, 108]. However, owing to the control of the COVID-19 epidemic in China, there has been a reduction in the number of patients, thereby restricting the number of studies evaluating the efficacy of TCM. To address this challenge, researchers are in the process of establishing animal models to further determine the efficacy of TCM in vivo. Apart from the lack of a large sample of randomized controlled clinical research data, the following urgent problems should be addressed to promote evidence-based Chinese medicine: the efficacy and safety of TCM lack scientific evidence; A rugged evaluation index system and evaluation methodology for disease prevention and treatment using TCM are currently lacking.

## Data Availability

Not applicable.

## Abbreviations

SARS-CoV-2: Severe acute respiratory coronavirus 2
COVID-19: Coronavirus disease 2019
TCM: Traditional Chinese medicine
WHO: World Health Organization
MERS: Middle east respiratory syndrome
ORF: Open reading frames
ACE2: Angiotensin-converting enzyme 2
CT: Computerized tomography
IL: Interleukin
GCSF: Granulocyte-colony stimulating factor
IP-10/CXCL10: IFN-γ-inducible protein 10
MCP-1/CCL2: Monocyte chemoattractant protein-1
MIP-1α/CCL3: Macrophage inflammatory protein-1α
MIP-1β: Macrophage inflammatory protein-1β
TNF-α: Tumor necrosis factor-α
ICU: Intensive care unit
RT-PCR: Real-time polymerase chain reaction
Ig: Immunoglobulin
EUA: Emergency use authorization
FDA: Food and Drug Administration
LH: Lianhua Qingwen
JH: Jinhua Qinggan
Mpro: 3CL hydrolase
XBJ: Xuebijing
XFBD: Xuanfeibaidu granules
